# Effects of *Usag-1* and *Bmp7* deficiencies on murine tooth morphogenesis

**DOI:** 10.1186/s12861-016-0117-x

**Published:** 2016-05-13

**Authors:** Kazuyuki Saito, Katsu Takahashi, Masakazu Asahara, Honoka Kiso, Yumiko Togo, Hiroko Tsukamoto, Boyen Huang, Manabu Sugai, Akira Shimizu, Masaharu Motokawa, Harold C. Slavkin, Kazuhisa Bessho

**Affiliations:** Department of Oral and Maxillofacial Surgery, Graduate School of Medicine, Kyoto University, Shogoin-Kawahara-cho 54, Sakyo-ku, Kyoto, 606-8397 Japan; College of Liberal Arts and Sciences, Mie University, Mie, Japan; School of Dentistry and Health Sciences, Sturt University, Orange, Australia; Department of Molecular Genetics, Division of Medicine, Faculty of Medical Sciences, University of Fukui, Fukui, Japan; Department of Experimental Therapeutics, Institute for Advancement of Clinical and Translational Science, Kyoto University Hospital, Kyoto, Japan; Kyoto University Museum, Kyoto University, Kyoto, Japan; Center for Craniofacial Molecular Biology, Division of Biomedical Sciences, Ostrow School of Dentistry, University of Southern California, Los Angeles, CA USA

**Keywords:** Tooth size, *Bmp7*, *Usag-1*, Mouse model, Tooth volume, Tooth morphogenesis

## Abstract

**Background:**

*Wnt5a* and *Mrfzb1* genes are involved in the regulation of tooth size, and their expression levels are similar to that of *Bmp7* during morphogenesis, including during the cap and early bell stages of tooth formation. We previously reported that *Usag-1*-deficient mice form supernumerary maxillary incisors. Thus, we hypothesized that BMP7 and USAG-1 signaling molecules may play important roles in tooth morphogenesis. In this study, we established double genetically modified mice to examine the in vivo inter-relationships between *Bmp7* and *Usag-1*.

**Results:**

We measured the volume and cross-sectional areas of the mandibular incisors using micro-computed tomography (micro-CT) in adult *Bmp7*- and *Usag-1*-LacZ knock-in mice and their F_2_ generation upon interbreeding. The mandibular incisors of adult *Bmp7*+/− mice were significantly larger than those of wild-type (WT) mice. The mandibular incisors of adult *Usag-1*−/− mice were the largest of all genotypes examined. In the F_2_ generation, the effects of these genes were additive; *Bmp7*+/− was most strongly associated with the increase in tooth size using generalized linear models, and the total area of mandibular supernumerary incisors of *Usag-1*−/−*Bmp7*+/− mice was significantly larger than that of *Usag-1*−/−*Bmp7* +/+ mice. At embryonic day 15 (E15), BrdU assays demonstrated that the labeling index of *Bmp7*+/− embryos was significantly higher than that of WT embryos in the cervical loop. Additionally, the labeling index of *Usag-1*−/− embryos was significantly the highest of all genotypes examined in dental papilla.

**Conclusions:**

*Bmp7* heterozygous mice exhibited significantly increased tooth sizes, suggesting that tooth size was controlled by specific gene expression. Our findings may be useful in applications of regenerative medicine and dentistry.

**Electronic supplementary material:**

The online version of this article (doi:10.1186/s12861-016-0117-x) contains supplementary material, which is available to authorized users.

## Background

Development of the dentition is regulated by time- and position-specific reciprocal epithelial-mesenchymal interactions [1–4]. These odontogenic interactions are directed and coordinated by transcription factors, growth and signaling factors, their cognate receptors, and extracellular matrix constituents [5]. Collectively, various combinations of molecules within these interactions determine when or where teeth develop and modulate the specifications for tooth size and shape.

*Noggin*, *Wnt5a*, and *Mfrzb1* genes have been shown to regulate tooth size [6–9]. Cai et al., using hetero-specific tissue recombination from rat molar tooth organs, demonstrated that tooth size is determined not by dental papilla mesenchymal cell number, but by intrinsic tissue-specific dental papilla mesenchymal factors [10]. In addition, exogenous treatment with bone morphogenic protein (BMP) 4, Noggin, fibroblast growth factor (FGF) 3, and FGF10 does not affect tooth size, despite the observation that BMP2/4, FGF3/10, LEF1, and WNT5a/5b are expressed in the dental mesenchyme during the cap and early bell stages of tooth morphogenesis [6, 11–15]. The crown width of a bioengineered molar reconstructed with dissociated epithelial and mesenchymal cells through the organ germ method is correlated with the length of the contact area between the epithelial and mesenchymal cell layers [16].

In conditional *Bmp7*-deficient embryos, the maxillary incisor tooth organs are either missing or hypoplastic, and the development of the first molar tooth organs is delayed, malformed, or missing [17]. Therefore, *Bmp7* is assumed to be an essential growth factor for tooth morphogenesis. BMP7 is a 35-kDa homodimeric protein that is associated with a variety of signaling pathways, including the canonical SMAD pathway, mitogen-activated protein kinase (MAPK)-related pathways, and the phosphoinositol 3-kinase (PI3K)/Akt pathway [18–21]. Uterine sensitization-associated gene-1 (USAG-1; also known as sclerostin domain-containing protein 1 [SOSTDC1], ectodin, and Wise) is a heavily glycosylated 28–30 kDa secretory protein that functions as a monomer to induce signal transduction. USAG-1 binds to the Wnt coreceptors low-density lipoprotein receptor-related protein (LRP) 5 and LRP6 and inhibits Wnt signaling [22, 23]. LRP4 modulates and integrates BMP and canonical Wnt signaling during tooth morphogenesis by binding to secreted USAG-1 [24]. USAG-1 expression is further restricted to the distal renal tubules, in a pattern similar to the localization of BMP7. USAG-1 is a BMP antagonist; it interacts with BMP7 in the developing and adult kidney and directly binds to BMP2/4/7, as assayed using co-immunoprecipitation studies [25]. Moreover, we previously reported that *Usag-1*-deficient mice exhibit supernumerary maxillary incisors in response to enhanced BMP signaling and that BMP signaling is modulated by Wnt signaling in *Usag-1*-deficient mice [26, 27].

Based on these observations and findings, we hypothesized that BMP7 and USAG-1 play important roles in tooth morphogenesis. Therefore, we previously established double genetically modified mice to analyze the in vivo inter-relationships between BMP7 and USAG-1. Using this model, we demonstrated that USAG-1 inhibits BMP7 signaling, leading to apoptosis and degeneration of rudimentary tooth germs in maxillary supernumerary incisor formation [28]. Moreover, we found that the tooth size in mandibular incisors of *Bmp7* heterozygous mice was altered. Accordingly, in this study, we examined the effects of BMP7 and USAG-1 signaling on tooth size in mandibular incisors using the F_2_ generation of mice.

## Methods

### Production and analysis of Usag-1- and Bmp7-LacZ knock-in mice

*Bmp7*-LacZ knock-in (ICR) mice and *Usag1*-LacZ knock-in (C57BL/6) mice were produced as previously described [29, 30]. *Bmp7*-deficient mice were embryonic lethal. Day E0 was established as midnight prior to finding a vaginal plug.

Polymerase chain reaction (PCR) amplification was performed using KOD FX NEO polymerase (TOYOBO, Osaka, Japan) and specific primers for genotyping.

### X-gal staining

The bacterial LacZ (β-galactosidase) gene in *Bmp7*LacZ/LacZ (ICR) and *Usag1*LacZ/LacZ (C57BL/6) mice was knocked into the gene of interest [29, 30]. Frozen sections of embryos were fixed in 4 % paraformaldehyde for 2 min and stained with X-Gal (Wako) twice as whole mounts and frozen sections, followed by counterstaining with Nuclear Fast Red (Kernechtrot). For X-gal staining, embryos were incubated at 37 °C in buffer (20 mg/mL X-gal/dimethylformamide, 35 mM K_3_Fe(CN)_6_, 35 mM K_4_Fe(CN)_6_, 2 mM MgCl_2_, 0.02 % NP-40, 0.01 % sodium deoxycholate, 1× phosphate-buffered saline [PBS]). We observed mandibular incisors in *Bmp7*+/− (ICR) and *Usag-1*+/− (C57BL/6) mice and mandibular molars in *Usag-1*+/− (C57BL/6) mice at E14 and E15.

### Analysis of the adult tooth phenotype

Three-dimensional (3D) computed tomography (CT) scans (SMX-100XT-SV3; Shimadzu, Kyoto, Japan) were performed using the heads of adult mice. We converted CB files to TIFF files. CB files had 512 × 512 pixels, 8 bits, and a voxel size of x:y:z = 1:1:1 (approximately 0.06 mm per side). Next, 3D images were reconstructed and analyzed with computer imaging software (INTAGE Realia and Volume Player software; KGT Inc, Tokyo, Japan) [31]. The mandibular incisor volume was measured using the imaging software; then, to measure the cross-sectional area, mandibular incisors were cut vertically to the tooth axes at the uppermost point on the incisor alveolar rim (at the bone-tooth junction), so as to avoid the influence of environmental factors, such as dental occlusion and tooth attrition. The cross-sectional area of the incisors was measured using ImageJ software. We analyzed a total of 22 wild-type (WT) and 20 *Bmp7*+/− samples in adult *Bmp7*-LacZ knock-in (ICR) mice at 2 months after birth, a total of 21 individual genotypes in adult *Usag1*LacZ knock-in (C57BL/6) mice at 3 months after birth, and nine *Usag-1*+/+*Bmp7*+/+, 12 *Usag-1*+/− *Bmp7*+/+, 18 *Usag-1*−/−*Bmp7*+/+, 14 *Usag-1*−/−*Bmp7*+/−, 28 *Usag-1*+/−*Bmp7*+/−, and 17 *Usag-1*+/+*Bmp7*+/− adult F_2_ generation mice at 4 months after birth. Data for volume and cross-sectional area obtained from the right and left incisors were summed.

We measured the cross-sectional area of mandibular supernumerary incisors of three *Usag-1*−/−*Bmp7*+/+ and six *Usag-1*−/−*Bmp7*+/− adult F_2_ generation mice. We used two *Usag-1*−/−*Bmp7*+/+ and six *Usag-1*−/−*Bmp7*+/− mice at 1 month after birth and one *Usag-1*−/−*Bmp7*+/+ mouse at 4 months after birth. The total area was measured in cases of multiple supernumerary teeth.

To evaluate the effects of *Bmp7* and *Usag-1* on the total dentition, we also measured mandibular molars. We obtained photographs of mandibular three molars of the F_2_ generation from directly above the occlusal view under a stereomicroscope and measured the projected area with ImageJ software using an image of a ruler taken at the same magnification. The distances between the lens and specimen were fixed. We examined unilateral mandibular molars from seven *Usag-1*+/+*Bmp7*+/+, 16 *Usag-1*+/−*Bmp7*+/+, 16 *Usag-1*+/+*Bmp7*+/−, and 31 *Usag-1*+/−*Bmp7*+/− adult F_2_ generation mice at 1 month after birth. Because molar form *Usag-1*−/− mice fused [24, 32, 33], we did not measure these molars.

### BrdU immunostaining

Cell proliferation was detected by BrdU immunostaining (BrdU Solution and BD Pharmingen BrdU In-situ Detection Kit; BD Biosciences) according to the manufacturer’s specifications. We made a working solution of BrdU in PBS at 1 mg/mL and injected the mice intraperitoneally with 1 mL of the BrdU solution. After 2 h, the mice were sacrificed, and the heads of the embryos were removed and fixed in 4 % paraformaldehyde overnight. Paraffin sections (7 μm) were then created. Background tissue was stained with hematoxylin for 5 s. We counted approximately 500–1000 nuclei under a light microscope, and the labeling index was determined as (BrdU+ cells/total nuclei)/100.

### Detection of apoptosis

Apoptotic cells in situ were detected by the TUNEL method using an apopTag Plus In Situ Apoptosis Detection Kit-Fluorescein (Chemicon International) according to the specifications of the manufacturer.

For paraffin-embedded sections, embryos were fixed in 4 % paraformaldehyde in PBS overnight. After fluorescent staining, the sections were counterstained with 4′,6-diamidino-2-phenylindole nuclear staining (Dapi Fluoromount-G; Southern Biotech).

### Statistical analysis

Data are presented as the means ± standard deviations. Statistical significance was assessed by analysis of variance (ANOVA) with the statistical program R. Gaussian distributions were determined by the Anderson Darling normality test.

In adult *Bmp7*-LacZ knock-in mice, 22 WT samples and 20 *Bmp7*+/− samples collected from mice at 2 months after birth were analyzed. We calculated the mean volume and area of the right and left incisors. Statistical significance was determined by unpaired one-tailed t-tests for volume and by Mann–Whitney U tests for cross-sectional area.

In adult *Usag1*-LacZ knock-in mice, 21 samples of individual genotypes at 3 months after birth were analyzed as stated above. Statistical significance was determined using Kruskal-Wallis and Steel-Dwass tests for multiple comparisons in volume and by one-way ANOVA using a Games Howell test for multiple comparisons in cross-sectional area.

In the adult F_2_ generation, we examined nine *Usag-1*+/+*Bmp7*+/+, 12 *Usag-1*+/−*Bmp7*+/+, 18 *Usag-1*−/−*Bmp7*+/+, 14 *Usag-1*−/−*Bmp7*+/−, 28 *Usag-1*+/−*Bmp7*+/−, and 17 *Usag-1*+/+*Bmp7*+/− mice, as mentioned above. Statistical significance was determined by one-way ANOVA using a Games Howell test for multiple comparisons.

In the analysis of mandibular molars in the adult F_2_ generation, statistical significance was determined by unpaired two-tailed t-tests (except for M1 and M2 in *Usag-1* +/− mice, for which Welch’s tests [M1] or Mann–Whitney U tests [M2] were used).

Finally, for analysis of cell proliferation using BrdU staining in the mandibular incisors in *Bmp7*-LacZ knock-in mice at E15 and analysis of mandibular supernumerary incisors in the adult F_2_ generation, statistical significance was determined using Mann–Whitney U tests. For analysis of cell proliferation using BrdU staining in the mandibular incisors in *Usag1*-LacZ knock-in mice at E15, statistical significance was determined using Kruskal-Wallis and Steel-Dwass tests for multiple comparisons.

## Results

### Expression of Bmp7 and Usag-1 at E14 and E15

**Fig. 1:**
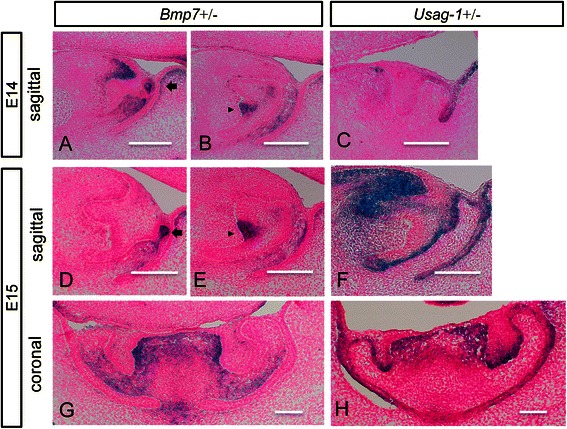
X-gal staining in mandibular incisors of *Bmp7*+/− (ICR) and *Usag-1*+/− (C57BL/6) mice at E14/E15. Tissue sections from mandibular incisors of *Bmp7*+/− (ICR) and *Usag-1*+/− (C57BL/6) mice at E14 and E15 were stained with X-gal (**a**–**h**). Scale bars indicate 100 μm. **a**–**f** Sagittal sections. **g**, **h** Coronal sections. **a**, **b**, **d**, **e**, **g** Embryos of *Bmp7*+/− (ICR) mice. *Bmp7* was expressed (blue) in the mesenchyme near the tooth germ. **c**, **f**, **h** Embryos of *Usag-1*+/− (C57BL/6) mice. *Usag-1* was expressed (blue) in a small portion of epithelia, except for the enamel knot and the mesenchyme near the tooth germ. **a**–**c** Embryonic day 14. **d**–**h** Embryonic day 15. **a**, **d** Sections near a rudimentary incisor (arrows) with strong expression are shown. **b**, **e** Sections near an enamel knot (arrowheads) with strong expression are shown

Sections from *Bmp7*+/− mice indicated that *Bmp7* was expressed (blue) in the mesenchyme around the tooth germ (Fig. 1a, d, g), rudimentary incisor (Fig. 1a, d), and enamel knot (Fig. 1b, e). Sections from *Usag-1*+/− mice indicated that *Usag-1* was expressed (blue) in a small portion of the epithelia, excluding the enamel knot and mesenchyme around the tooth germ at E15, during the early bell stage (Fig. 1f, h,) (Additional file 1: Figure S1B, C).

### Micro-CT analysis of the mandibular incisors in adult Bmp7-LacZ knock-in mice

**Fig. 2:**
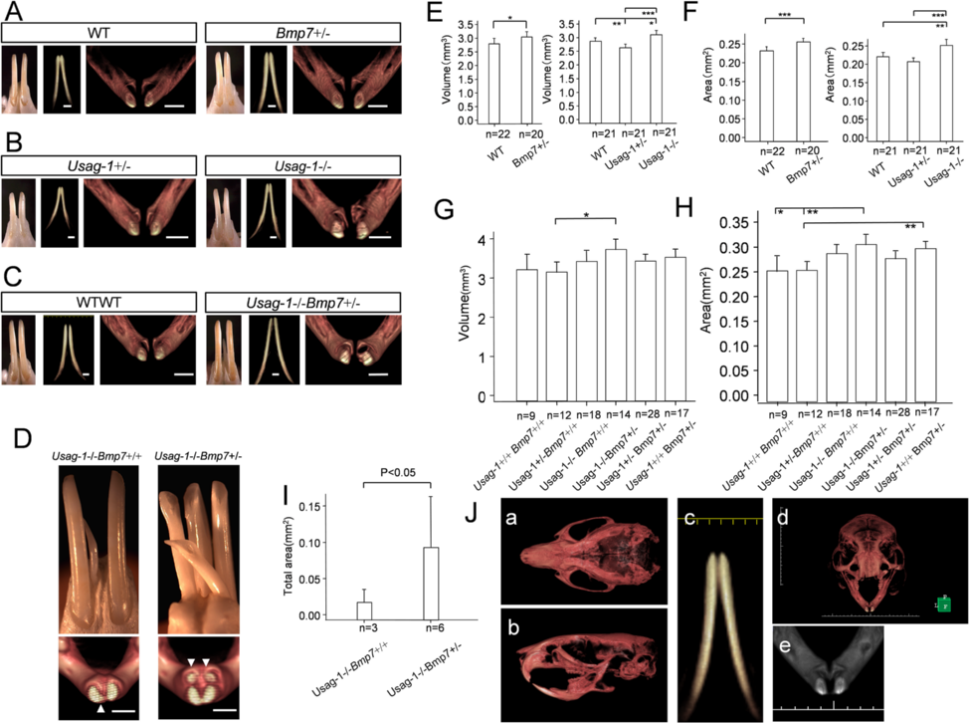
Analysis of the adult incisor phenotype. **a** Micro-CT analysis of mandibular incisors at 2 months after birth. Left to right: image from a stereomicroscope, three-dimensional image, and cross-section. **b** Micro-CT analysis of mandibular incisors at 3 months after birth. Left to right: image from a stereomicroscope, three-dimensional image, and cross-section. **c** Left to right: image from a stereomicroscope, three-dimensional image, and cross-section. **d** Upper images are from a stereomicroscope. Left image is the lingual side. Right image is the buccal side. Lower images show the cross-sectional area of mandibular supernumerary teeth (arrowheads) and mandibular incisors. **a**–**d** Scale bars: 1 mm. **e**, **f** Volume and cross-sectional area of adult mandibular incisors. Numbers indicate respective incisors. Data in (**e**) are the mean volume ± standard deviation (SD) of mandibular incisors at 2 months (left) or 3 months (right) after birth. Data in (**f**) are the mean cross-sectional area ± SD of mandibular incisors at 2 months (left) or 3 months (right) after birth. **g**, **h** Volume and cross-sectional area of mandibular incisors in the adult F_2_ generation at 4 months after birth. Data in (**g**) are the mean volume ± SD of mandibular incisors. Data in (**h**) are the mean cross section ± SD of mandibular incisors. **I** Statistical analysis of cross-sectional area of mandibular supernumerary incisors in the adult F_2_ generation at 1 month. Error bars: SDs. **j** Images of trimmed and extracted mandibular incisors in **a**, **b**, and **c**. Whole computed tomography images of a mouse skull bone and an incisor with soft tissue removed using INTAGE Realia are shown (*a*, *b*, and *c*). We opened the image in Volume Player software (KGT Inc., Tokyo, Japan) (*d*), created .bmp files, and opened the files in Image J software (*e* ). *P < 0.05, **P < 0.01, ***P < 0.001

The volume and cross-sectional area of the mandibular incisors were larger in *Bmp7*+/− (ICR) mice than in WT mice (Fig. 2a). Volumes of *Bmp7*+/− mice were significantly larger than those of WT mice (Fig. 2e). The cross-sectional areas of *Bmp7*+/− mice were significantly larger than those of WT mice (Fig. 2f).

### Micro-CT analysis of the mandibular incisors in adult Usag1-LacZ knock-in mice

The volume and cross-sectional area of the mandibular incisors in *Usag-1*LacZ knock-in (C57BL/6) mice were larger than those in any other diplotype (Fig. 2b). The volume and cross-sectional area in *Usag-1*−/− mice were significantly larger than those in all other diplotype (Fig. 2e, f). The volume and cross-sectional area in *Usag-1*+/− mice were significantly smaller than those in WT mice (Fig. 2e, f).

Few mice exhibited malocclusion among *Usag-1*−/− mice. Mice that had malocclusion, taking into account feeding disorders and changes in tooth size, were excluded from the statistical analyses. Two of 23 *Usag-1*−/− mice were excluded (Additional file 2: Figure S2C),

### Analysis of the projected areas of the occlusal surface of mandibular molars in adult F_2_ mice

**Fig. 3:**
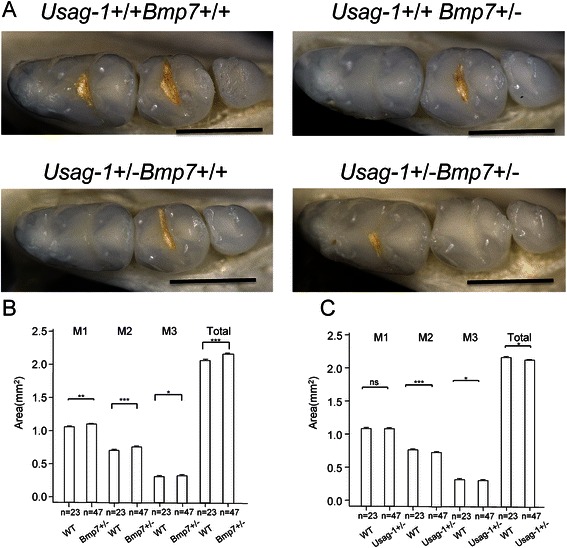
Analysis of the adult molar phenotype. **a** Occlusal surface of mandibular molars in the F_2_ generation at 1 month after birth. Scale bar indicates 1 mm. **b**, **c** Statistical analysis of projected areas of the occlusal surface of three molars and all mandibular molars of the F_2_ generation at 1 month after birth. ns: not significant, *P < 0.05, **P < 0.01, ***P < 0.001. **b** Mean area ± standard deviation (SD) of mandibular molars in WT (*n* = 23) and *Bmp7*+/− (*n* = 47) mice. **c** Mean area ± standard deviation (SD) of mandibular molars in WT (*n* = 23) and *Usag-1*+/− (*n* = 47) mice

The areas of the occlusal surface of the mandibular molars in *Bmp7*+/− mice were significantly larger than those in WT mice for both the three individual molars and the total (Fig. 3a, b). The areas of the occlusal surface of the mandibular molars in *Usag-1*+/− mice were significantly smaller than those in WT mice, except for M1 (Fig. 3a, c).

We used a generalized linear model (GLM) to examine the effects of respective genes and genotypes on tooth size. The model formula of the GLM was “Tooth Size = *Usag-1* + *Bmp7*”. We did not consider interactions. From this analysis, we found that *Bmp7*+/− was associated with an increase in tooth size, whereas *Usag-1*+/− was associated with a decrease in area (Table 1). The error structure of the response variable followed a Gaussian distribution. The link function was a linear model. The explanatory variable was categorical data. We used genotypes as the categorical data.

**Table 1:** A generalized linear model to analyze adult tooth size. The response variable is the total area of the occlusal surface of the molars of the F_2_ generation at 1 month after birth

	Estimate	Std.Error	T value	*P* value
*Usag-1*+/−	−0.05311	0.02503	−2.122	0.03755
*Bmp7*+/−	0.09814	0.02503	3.921	0.00021

### Micro-CT analysis of the mandibular incisors in the adult F_2_ generation

The volume and cross-sectional area of the mandibular incisors of *Usag-1*−/−*Bmp7*+/− mice were larger than those of all other genotypes (Fig. 2c). This analysis clarified that the effects of each diplotype of *Bmp7* and *Usag-1* were additive for volume and cross-sectional area (Fig. 2g, h).

Next, a GLM was used, as described in the Methods. *Usag-1*−/− and *Bmp-7*+/− were associated with increased tooth size, whereas *Usag-1*+/− was associated with decreased tooth size (Table 2). This analysis confirmed that *Bmp7*+/− was most strongly associated with an increase in tooth size.

**Table 2:** A generalized linear model to analyze adult tooth size. The response variables are volume and cross-section of the mandibular incisors of the F_2_ generation at 4 months after birth

	Volume				Area			
	Estimate	Std.Error	T value	*P* value	Estimate	Std.Error	T value	*P* value
*Usag-1*+/−	−0.08545	0.11291	−0.757	0.45107	−0.012964	0.008759	−1.480	0.142169
*Usag-1*−/−	0.20793	0.12003	1.732	0.08651	0.019976	0.009311	2.145	0.034498
*Bmp7*+/−	0.29578	0.09514	3.109	0.00249	0.027771	0.007380	3.763	0.000292

Two of 16 *Usag-1*−/−*Bmp7*+/− mice exhibited malocclusion was excluded (Additional file 2: Figure S2D),

### Analysis of cell proliferation using BrdU staining of the mandibular incisors at E15

In murine incisors and molars, tooth eruption and tooth root formation are completed by about P20 or P21. The cap stage and early bell stage are considered equal to the morphogenetic phase. During the early bell stage, only the labial epithelium gives rise to the enamel-forming ameloblasts in mandibular incisors [34]. Murine incisors are continuously growing; however, *Bmp7* and *Usag-1* are not expressed in enamel epithelial stem cells in adult mice [35].

**Fig. 4:**
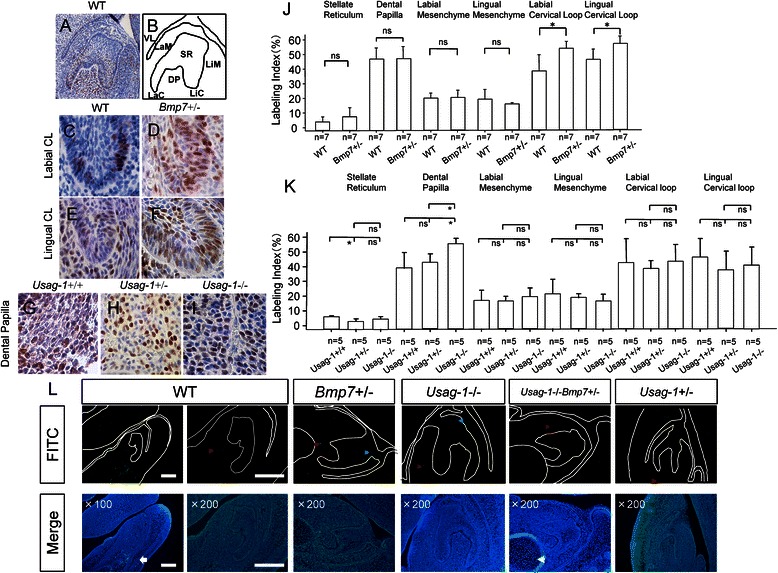
BrdU immunostaining and detection of apoptosis in mandibular incisors at E15. **a**–**j** Analysis of proliferation using BrdU staining in mandibular incisors at E15. **a** Sagittal section of mandibular incisors (200× magnification) of WT mice. **b** Schematic diagram. VL: vestibular lamina, SR: stellate reticulum, DP: dental papilla, LaM: labial mesenchyme, LiM: lingual mesenchyme, LaC: labial cervical loop, LiC: lingual cervical loop. **c**–**f** Sagittal section of cervical loop (1000× magnification). (G-I) Sagittal section of dental papilla (1000× magnification). **j** Analysis of cell proliferation using BrdU staining in mandibular incisors of WT and *Bmp7*+/− (ICR) mice at E15. Percent labeling index in respective tissues of WT and *Bmp7*+/− E15 embryos are shown. Labeled cells were counted under 1000× magnification. We counted approximately 500 nuclei under a light microscope and determined the labeling index as: (BrdU+ cells / total nuclei) / 100. Data are the mean + standard deviation (SD). ns: not significant, *P < 0.01 by Mann–Whitney U test. **k** Analysis of cell proliferation using BrdU staining in mandibular incisors of *Usag-1*+/+, *Usag-1*+/− and *Usag-1* −/− (C57BL/6) mice at E15. Percent labeling index of mandibular incisors of *Usag-1*+/+, *Usag-1*+/− and *Usag-1*−/− embryos at E15. Data are the mean ± SD. ns: not significant, *P < 0.05 by Steel Dwass test. **l** TUNEL staining in mandibular incisors of *Bmp7*+/− (ICR), *Usag-1*−/− (C57BL/6), *Usag-1*−/−*Bmp7*+/− (F_2_), and *Usag-1*+/− (C57BL/6) mice at E15. Under 100× magnification, the developing bone and cartilage (white arrow) are shown. Under 200× magnification, Meckel’s cartilage (white arrowhead), epithelium near the oral cavity (green arrowheads), and the mesenchyme near the tooth germ (red arrowheads) are shown. All bars: 100 μm

Therefore, we investigated cell proliferation by BrdU staining in the mandibular incisors at E15, during the early bell stage. The tissues of the mandibular incisors at the early bell stage were categorized as vestibular lamina, stellate reticulum, dental papilla, labial mesenchyme, lingual mesenchyme, labial cervical loop, and lingual cervical loop (Fig. 4a, b). The labeling index of *Bmp7*+/− embryos was significantly higher than that of WT embryos in the cervical loop (Fig. 4c–f, j). Additionally, the labeling index of *Usag-1*−/− embryos was significantly higher than that of WT and *Usag-1*+/− embryos in dental papilla (Fig. 4g-i, k). The labeling index of WT embryos was significantly higher than that of *Usag-1*+/− embryos in stellate reticulum (Fig. 4k).

### TUNEL staining in the mandibular incisors at E15

Next, we investigated the occurrence of apoptosis in cells in the mandibular incisors of *Bmp7*+/− (ICR), *Usag-1*−/− (C57BL/6), *Usag-1*−/−*Bmp7*+/− (F_2_ generation), and *Usag-1*+/− (C57BL/6) embryos at E15 (Fig. 4l). Apoptotic cells were detected near Meckel’s cartilage, the developing bone and cartilage, the epithelium near the oral cavity, and the mesenchyme near the tooth germ (Fig. 4l). Few apoptotic cells were observed in the tooth germ for all genotypes. In the early bell stage, apoptosis was not associated with the determination of tooth size.

### Analyzing mandibular supernumerary incisors in the adult F_2_ generation

In the F_2_ generation, the size of supernumerary incisors of *Usag-1*−/−*Bmp7*+/− mice was larger than that of *Usag-1*−/−*Bmp7*+/+ mice (Fig. 2d). Moreover, the total area of mandibular supernumerary incisors of *Usag-1*−/−*Bmp7*+/− mice was significantly larger than that of *Usag-1*−/−*Bmp7*+/+ mice (Fig. 2i).

## Discussion

The expression levels of *Usag-1* and *Bmp7* are opposing in the region near the rudimentary incisor tooth primordia between the maxilla and mandible [28]. Moreover, in several types of genetically modified mice, the dental phenotype differs between the maxilla and mandible [13, 36, 37]. For example, in *Usag-1*-deficient mice, supernumerary teeth are observed in 100 % of the maxillary incisor regions, whereas partial penetrance is observed in the mandible [26]. Thus, the genetic controls for tooth organ size and shape play critical roles in tooth regeneration. In this report, we demonstrated that the sizes of mandibular incisor and molar tooth organs in *Bmp7*+/− mice were significantly larger than those of WT mice. In contrast, the sizes of incisors and molars were significantly smaller in *Usag-1*+/− mice than in WT mice, and the volume and cross-sectional areas in *Usag-1*−/−, mice were significantly larger than those of all other genotypes. Thus, our results demonstrated, for the first time, that *Bmp7* and *Usag-1* heterozygous mice exhibited changes in tooth size.

In conditional *Bmp7*-deficient embryos, the maxillary incisors have been shown to be missing or hypoplastic [17]. Thus, the phenotypes of *Usag-1* heterozygous mice and *Bmp7* heterozygous mice were opposite those of *Usag-1*-deficient mice and *Bmp7*-deficient mice, respectively. Our results suggested that the levels of *Bmp7* and *Usag-1* expression in heterozygotes did not reach the threshold level necessary for normal tooth morphogenesis, that is, the embryos exhibited haploinsufficiency. Furthermore, heterozygotes may exhibit changes in downstream gene expression, resulting in changes in tooth size. Global analysis of gene expression in heterozygotes, such as microarray analysis and next-generation sequencing, is needed in order to elucidate the molecular mechanism involved in these processes.

The expression level of *Bmp7* was similar to those of *Wnt5a* and *Mrfzb1* and was localized adjacent to *Usag-1* expression in the dental mesenchyme. USAG-1 is a BMP7 antagonist; however, the effects of BMP7 and USAG-1 were additive within the mandibular incisors of the F_2_ generation. BrdU assays confirmed that cell proliferation was increased within the cervical loop in association with larger sized mandibular incisors in *Bmp7*+/− embryos. Furthermore, we confirmed that the increased cell proliferation of dental papilla mesenchymal cells was associated with larger mandibular incisors in *Usag-1*−/− embryos. Importantly, apoptosis was not associated with tooth size at E15. These data indicated that there was a difference in the mechanisms of action of BMP7 and USAG-1 during the cap or bell stages of tooth morphogenesis. Continuous growth and enamel deposition in incisors can be modulated by the levels of FGF3/10, activin, and BMP2/4/7 mesenchymally expressed in the epithelial stem cell niche [38]. Thus, we concluded that elongation of the cervical loop in *Bmp7*+/− embryos at E15 enlarged the incisors and that BMP7 expressed in the mesenchyme around the cervical loop had distinct local effects on the loop.

The volume and cross-sectional area were largest in *Usag-1*−/− mice, and the labeling index of the dental papilla in *Usag-1*−/− mice was highest of all genotypes examined. We have also reported that phosphorylated SMAD1/5/8 levels are increased and that β-catenin is localized in the nucleus in odontogenic mesenchymal cells within the maxillary rudimentary incisor tooth organ in *Usag-1*-deficient embryos [27]. Using organ culture of WT and *Usag-1*-deficient mandibular incisors, Munne et al. demonstrated that *Usag-1* expression is limited to the mesenchyme and that the dental mesenchyme may limit supernumerary tooth induction resulting from activated Wnt signaling, thereby minimizing the amount of mesenchymal tissue surrounding the incisor tooth germs prior to culture [39]. USAG-1 is downstream of sonic hedgehog (Shh) signaling; therefore, a Wnt-Shh-SOSTDC1 negative feedback loop may control the spatial patterning of teeth, and Wnt, Shh, and SOSTDC1 may act as the activator, mediator, and inhibitor, respectively, in reaction–diffusion models [32]. Consistent with this, patients with supernumerary teeth have larger teeth than controls in humans [40]. Thus, these findings, combined with our new results, suggest that enhanced Bmp- and Wnt*-*mediated signal transduction in the dental mesenchyme of *Usag-1*−/− mice may increase the proliferation of cells in the dental mesenchyme at the cap or early bell stages, resulting in increased tooth size and formation of supernumerary teeth.

The size of a tooth is determined not only by genes related to tooth development but also by the development of the mandible. The size of mandibular incisors is altered by craniosynostoss in the Apert *Fgfr2*^S252W^ mouse model [41]. Importantly, in our study, we found no significant difference in the linear distance of the anteroposterior diameter and altitude between respective genotypes in *Bmp7*-LacZ knock-in mice at 2 months of age (Additional file 3: Figure S3B). However, at 3 months of age, *Usag-1*−/− mice exhibited a significantly longer linear distance of the anteroposterior diameter and altitude compared with that in WT and *Usag-1*+/− mice (Additional file 3: Figure S3C). The development of the mandible in the context of *Usag-1* deficiency may be associated with changes in the size of the mandibular incisor and molar in *Usag-1*−/− mice. These results suggested that we may be able to control the size of teeth by regulating the gene expression level locally in humans when dental epithelial stem cells, such as third dentitions and outer enamel epithelium, are used for tooth regeneration in vivo.

## Conclusion

Our findings showed that *Bmp7* heterozygous mice exhibited dramatic increases in tooth size and that tooth size was controlled by the expression levels of specific genes.

## Ethics approval and consent to participate

The study protocol and procedures were approved by the Animal Research Committee of Kyoto University (reference number: Med Kyo 11518) and the Recombinant DNA Experiment Safety Committee of Kyoto University. All experiments were carried out in accordance with the approved guidelines.

## Consent for publication

Not applicable.

## Additional files

Additional file 1: Figure S1. X-gal staining in mandibular molars of *Usag-1*+/− (C57BL/6) mice at E14 and E15. Tissue sections from mandibular molars of *Usag-1*+/− (C57BL/6) mice at E14 and E15 were stained with X-gal. Scale bar: 100 μm. (A, B) Sagittal sections. (C) Coronal sections. (B, C) *Usag-1* was expressed (blue) in a small portion of epithelia, except for the enamel knot and the mesenchyme near the tooth germ. (TIF 16106 kb) Additional file 2: Figure S2. Malocclusion observed in *Usag-1*−/− (C57BL/6) mice and *Usag-1*−/− *Bmp7*+/− (F_2_ generation) mice. (A) Normal occlusion. A *Usag-1*−/− female mouse in the C57BL/6 background at 3 months after birth. These data were added in the analysis of the lower incisors. Scale bar: 1 mm. (B) Normal occlusion. A *Usag-1*−/−*Bmp7*+/− male mouse in the F_2_ generation at 4 months after birth. These data were added in the analysis of the lower incisors. (C) Malocclusion. A *Usag-1*−/− female mouse in C57BL/6 background at 3 months after birth. The mouse was excluded from the analysis of the lower incisors. (D) Malocclusion. A *Usag-1*−/−*Bmp7*+/− female mouse in the F_2_ generation at 4 months after birth. The mouse was excluded from the analysis of the lower incisors. (TIF 14671 kb) Additional file 3: Figure S3. Difference in mandibular morphology between respective genotypes in *Bmp7* or *Usag-1*-LacZ knock-in mice. (A) Wild-type mouse (ICR) mandible at 2 months after birth with locations of landmarks used to analyze the morphological differences between respective genotypes. Linear distances between the identifiable landmarks were measured. a: inferior-most point on the incisor alveolar rim, b: anterior point on the molar alveolar rim, c: inferior-most point on border of the ramus inferior to incisor alveolar, d: mandibular angle. (B) Difference in mandibular morphology between WT and *Bmp7*+/− mice. We analyzed a total of 6 WT and 6 *Bmp7*+/− samples in adult *Bmp7*-LacZ knock-in (ICR) mice at 2 months after birth. Statistical significance was determined by the Mann–Whitney U test. (C) Difference in the mandibular morphology among WT, *Usag-1*+/−, and *Usag-1*−/− mice. We analyzed a total of 6 individual genotypes in adult *Usag1*-LacZ knock-in (C57BL/6) mice at 3 months after birth. Statistical significance was determined using a Kruskal-Wallis test and a Steel-Dwass test for multiple comparisons. **P* < 0.05. (TIF 3530 kb)

## Abbreviations

−/−: Knockout genotype
+/−: Heterozygous genotype
+/+: Wild-type
LacZ/LacZ: Knockout genotype in LacZ knock-in mice
WT: Wild-type
