# Supplementary figures and images for: Effects of Usag-1 and Bmp7 deficiencies on murine tooth morphogenesis
Source: BMC Dev Biol. 2016 May 13;16:14. doi: 10.1186/s12861-016-0117-x (PMC4866418; doi:10.1186/s12861-016-0117-x)

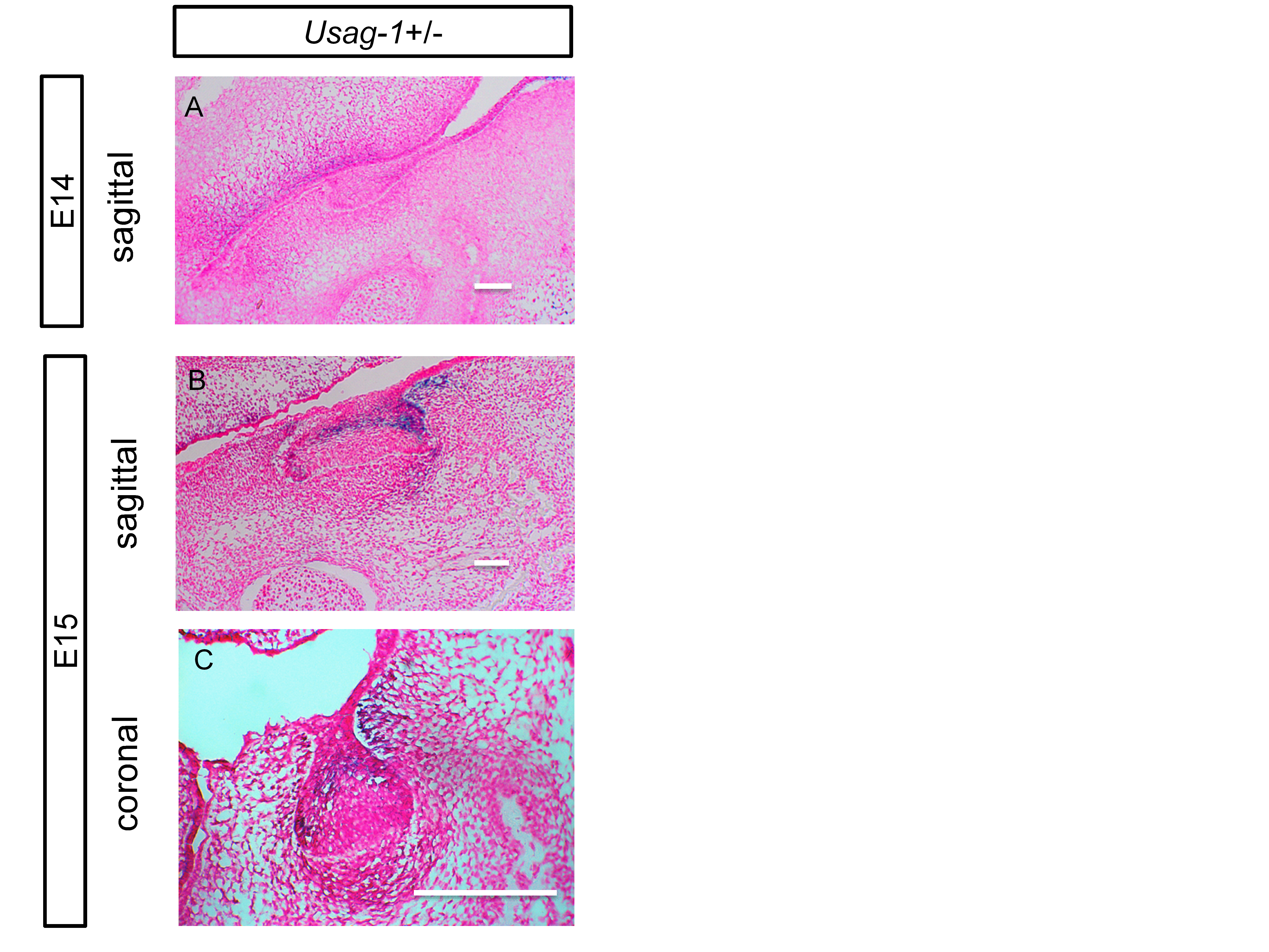

Supplement: Additional file 1: Figure S1. — X-gal staining in mandibular molars of Usag-1+/− (C57BL/6) mice at E14 and E15. Tissue sections from mandibular molars of Usag-1+/− (C57BL/6) mice at E14 and E15 were stained with X-gal. Scale bar: 100 μm. (A, B) Sagittal sections. (C) Coronal sections. (B, C) Usag-1 was expressed (blue) in a small portion of epithelia, except for the enamel knot and the mesenchyme near the tooth germ. (TIF 16106 kb) [file 12861_2016_117_MOESM1_ESM.tif]

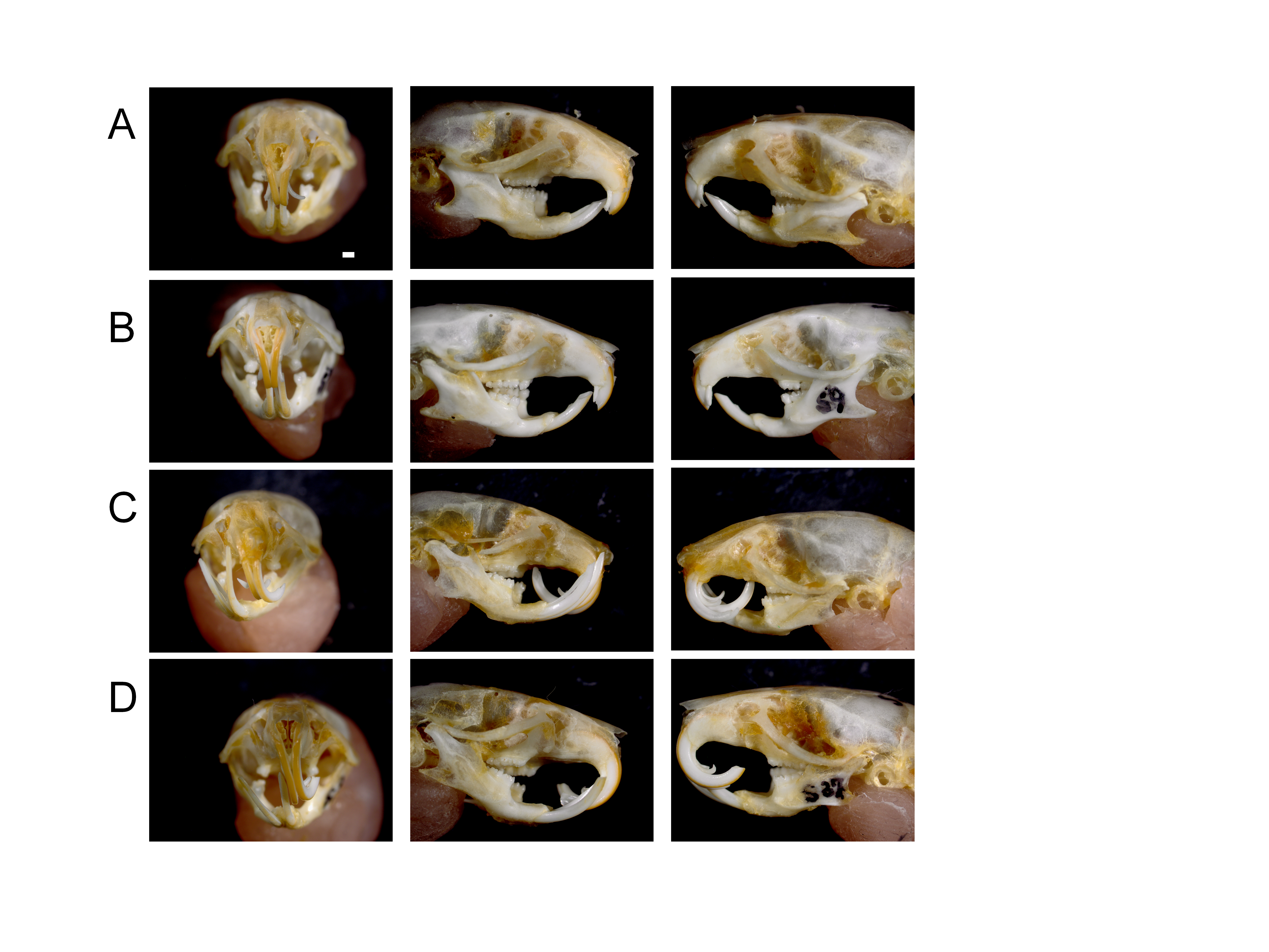

Supplement: Additional file 2: Figure S2. — Malocclusion observed in Usag-1−/− (C57BL/6) mice and Usag-1−/− Bmp7+/− (F2 generation) mice. (A) Normal occlusion. A Usag-1−/− female mouse in the C57BL/6 background at 3 months after birth. These data were added in the analysis of the lower incisors. Scale bar: 1 mm. (B) Normal occlusion. A Usag-1−/−Bmp7+/− male mouse in the F2 generation at 4 months after birth. These data were added in the analysis of the lower incisors. (C) Malocclusion. A Usag-1−/− female mouse in C57BL/6 background at 3 months after birth. The mouse was excluded from the analysis of the lower incisors. (D) Malocclusion. A Usag-1−/−Bmp7+/− female mouse in the F2 generation at 4 months after birth. The mouse was excluded from the analysis of the lower incisors. (TIF 14671 kb) [file 12861_2016_117_MOESM2_ESM.tif]

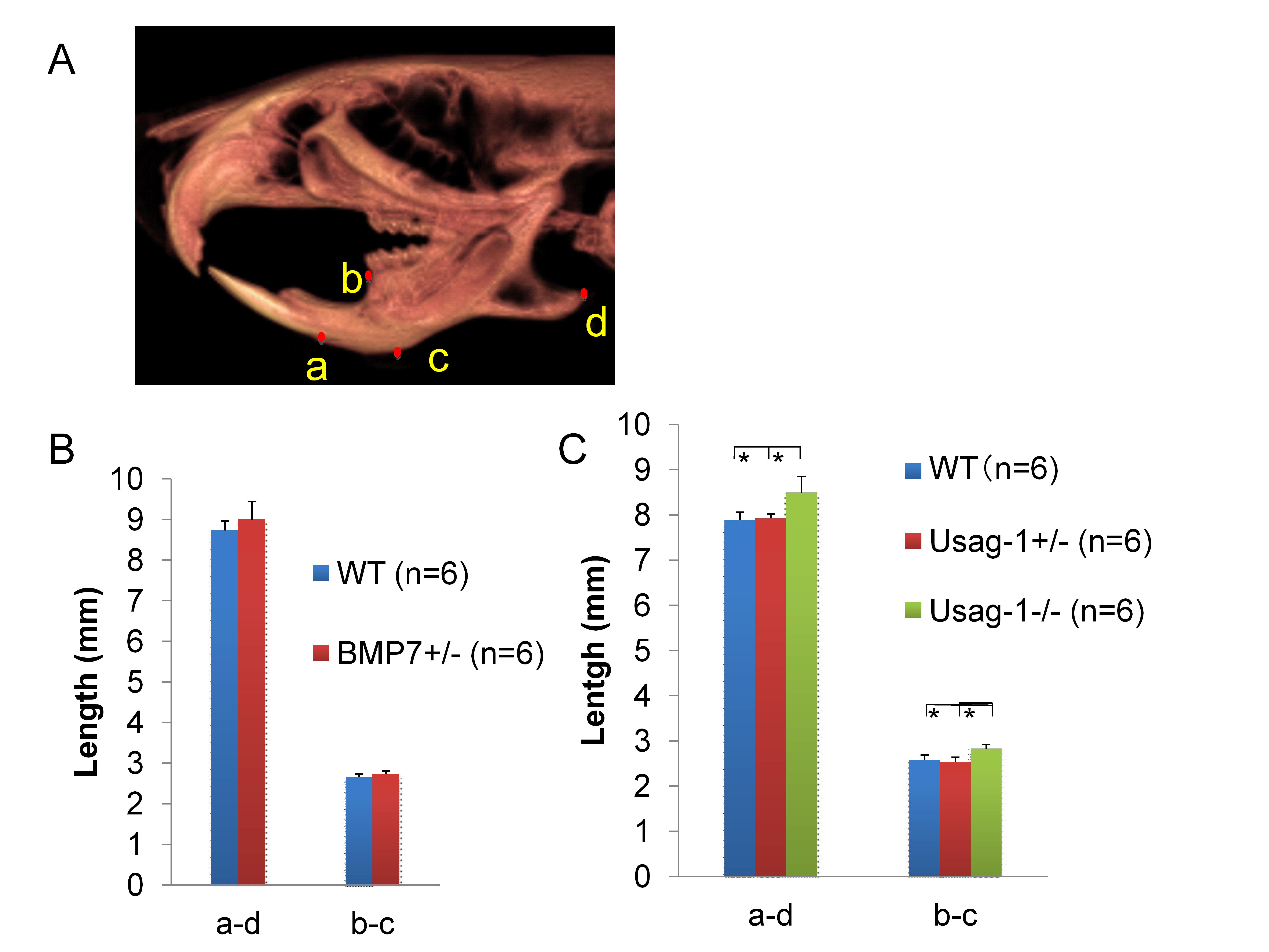

Supplement: Additional file 3: Figure S3. — Difference in mandibular morphology between respective genotypes in Bmp7 or Usag-1-LacZ knock-in mice. (A) Wild-type mouse (ICR) mandible at 2 months after birth with locations of landmarks used to analyze the morphological differences between respective genotypes. Linear distances between the identifiable landmarks were measured. a: inferior-most point on the incisor alveolar rim, b: anterior point on the molar alveolar rim, c: inferior-most point on border of the ramus inferior to incisor alveolar, d: mandibular angle. (B) Difference in mandibular morphology between WT and Bmp7+/− mice. We analyzed a total of 6 WT and 6 Bmp7+/− samples in adult Bmp7-LacZ knock-in (ICR) mice at 2 months after birth. Statistical significance was determined by the Mann–Whitney U test. (C) Difference in the mandibular morphology among WT, Usag-1+/−, and Usag-1−/− mice. We analyzed a total of 6 individual genotypes in adult Usag1-LacZ knock-in (C57BL/6) mice at 3 months after birth. Statistical significance was determined using a Kruskal-Wallis test and a Steel-Dwass test for multiple comparisons. *P < 0.05. (TIF 3530 kb) [file 12861_2016_117_MOESM3_ESM.tif]
